# Natural and Derivative Brevetoxins: Historical Background, Multiplicity, and Effects

**DOI:** 10.1289/ehp.7499

**Published:** 2005-02-10

**Authors:** Daniel G. Baden, Andrea J. Bourdelais, Henry Jacocks, Sophie Michelliza, Jerome Naar

**Affiliations:** University of North Carolina at Wilmington, Center for Marine Science, Wilmington, North Carolina, USA

**Keywords:** brevenal, brevetoxin, Florida red tide, immunotoxicant, *Karenia brevis*, neurotoxin, PbTx, polyether, pulmonary toxicant

## Abstract

Symptoms consistent with inhalation toxicity have long been associated with Florida red tides, and various causal agents have been proposed. Research since 1981 has centered on a group of naturally occurring *trans*-fused cyclic polyether compounds called brevetoxins that are produced by a marine dinoflagellate known as *Karenia brevis*. Numerous individual brevetoxins have been identified from cultures as well as from natural bloom events. A spectrum of brevetoxin derivatives produced by chemical modification of the natural toxins has been prepared to examine the effects of functional group modification on physiologic activity. Certain structural features of natural and synthetic derivatives of brevetoxin appear to ascribe specific physiologic consequences to each toxin. Differential physiologic effects have been documented with many of the natural toxins and derivatives, reinforcing the hypothesis that metabolism or modification of toxin structures modulates both the specific toxicity (lethality on a per milligram basis) and potentially the molecular mechanism(s) of action. A series of naturally occurring fused-ring polyether compounds with fewer rings than brevetoxin, known as brevenals, exhibit antagonistic properties and counteract the effects of the brevetoxins in neuronal and pulmonary model systems. Taken together, the inhalation toxicity of Florida red tides would appear to depend on the amount of each toxin present, as well as on the spectrum of molecular activities elicited by each toxin. Toxicity in a bloom is diminished by the amount brevenal present.

## Progress on Toxin Structural Character

Florida red tide inhalation toxicity has been documented in the Western literature since 1844. Earlier accounts from Spanish explorers of an “irritating essence” predate 1600. Past explanations for Florida red tide respiratory distress have included World War I nerve gas release (Galtsoff 1948), “fast”- and “slow”-acting toxins (McFarren et al. 1965), a phosphorylated organic molecule (Martin and Chatterjee 1969), and green particles as fragments of a marine alga (Woodcock 1948). Descriptions of Florida red tide complexity have mirrored increases in capability of analytical detection. Before 1970, the description of red tides was based on environmental observations of irritating aerosols, fish-killing substances, and hemolytic factors [reviewed in Baden (1983, 1989)]. Because such small amounts of bioactive material were necessary to elicit the toxicologic responses, much of the initial pharmacology/toxicology was understood long before toxin structures were known. Investigators in the early 1970s, using the new field of high-performance liquid chromatography, purified two toxins with very similar spectroscopic characteristics, their differences being the presence of either an exomethylene-conjugated aldehyde or the corresponding allylic alcohol. These two materials of “interchangeable nature” (T46 and T47, or T17 and T34) later became known as the polyether brevetoxins PbTx-3 and PbTx-2, respectively (Baden 1977; Catterall and Risk 1981; Poli et al. 1986). The structure of PbTx-2 (brevetoxin B) was determined by Nakanishi’s group in 1981 to be a *trans*-fused polyether ladder toxin with *syn* relative stereochemistry across each side of the molecule (Lin et al. 1981). Additional toxins were structurally characterized by comparison with the PbTx-2 spectroscopic data; PbTx-3 was proposed as the reduced form of PbTx-2 by Baden and Mende (1981) and Chou and colleagues (Chou et al. 1985; Shimizu et al. 1990). Subsequent reduction of PbTx-2 with tritium-labeled sodium borohydride allowed Baden and colleagues to produce isotopically labeled brevetoxin with sufficient specific activity for radioimmunoassays (Baden et al. 1984) and later to characterize the brevetoxin binding site in rat brain synaptosome sodium channels (Poli et al. 1986).

**Figure 1:**
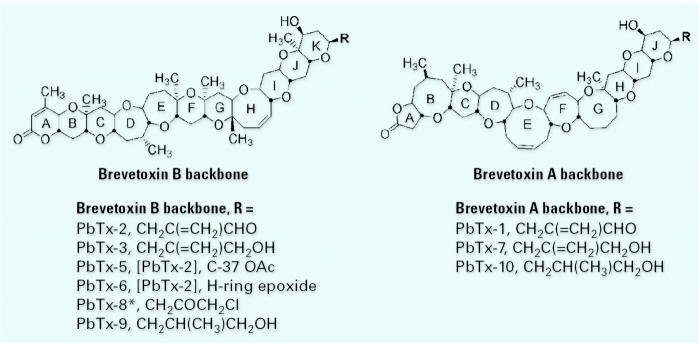
Brevetoxins are based on two different structural backbones, based on what are perceived to be the two parent molecules, PbTx-2 (brevetoxin B) and PbTx-1 (brevetoxin A). All other known derivatives are based on alteration of the R-side chain, epoxidation across the double bond in the H-ring of PbTx-2, or derivatization at the C-37 hydroxyl in PbTx-2. PbTx-8, the chloromethyl ketone derivative of PbTx-2, is an artifact of chloroform extraction and subsequent phosgene conversion of PbTx-2. Common features include *trans*-fused polyether ring systems consisting of five- to nine-membered rings.
*Denotes likely chemical artifact from extraction.

Shimizu et al. (1986) described brevetoxin A, possessing a slightly different polyether backbone, from cultures of *Karenia brevis*. This molecule, denoted PbTx-1, is very similar to the PbTx-2 structure in that both possess lactone functionality in the A-ring and a series of relatively rigid rings that form a ladder structure, and both terminate in an identical, very reactive α, β-unsaturated aldehyde side chain. It is noteworthy that PbTx-1 is the only natural toxin known to possess rings with five, six, seven, eight, and nine members, all in the same molecule. Seven additional structures followed in quick succession (Figure 1), the structures being determined by comparison of spectra with those of the two parent toxins, PbTx-1 and PbTx-2.

## Toxin Structure–Activity Relationships

By molecular modeling, Rein et al. (1994a, 1994b) and Gawley et al. (1995) determined that the structures of both PbTx-1 and PbTx-2 are relatively linear with a bend approximately mid-molecule, possess a lactone functionality in the A-ring, have a strictly rigid region in the terminal four rings, possess a side chain allowing modest modification at the molecules’ termini, and have a spacer region that separates the rigid region from the A-ring lactone. All natural brevetoxins and synthetic derivatives with full activity possess all these features. Numerous publications describe many of the salient features of the brevetoxin molecules and cite the modulating effects of altering the lactone functionality, of changing the nature of the side chain, of inducing additional flexibility in the middle of the molecule, or combinations thereof [e.g., Jeglitsch et al. (1998); Purkerson et al. (1999)]. All derivatives of natural toxin examined to date reduce toxicity; that is, no derivative has been produced synthetically that is more toxic than PbTx-1 or PbTx-2, the toxins reputed to be the parent molecules. In fact, it can be argued that all other natural toxins are produced biosynthetically by *K. brevis* using the two parent backbones as precursors.

Nicoloau et al. (1995) had produced totally synthetic brevetoxin PbTx-2, which had receptor binding properties and spectroscopy identical to the natural material, confirming that the structure proposed by Lin et al. (1981) was indeed correct. Subsequent synthesis of the brevetoxin A molecule (PbTx-1) was likewise accomplished (Nicoloau et al. 1998). The production of truncated brevetoxin by Nicoloau and colleagues, a compound possessing all of the “required” features of intact brevetoxin except for the B–E ring spacer region, was essentially inactive in patch-clamp experiments (Gawley et al. 1995), although it had some affinity for receptor site 5 on the sodium channel.

As a result of the multiplicity of effects demonstrated by this combination of metabolically relevant structural modifications, receptor binding pharmacology, and single-channel kinetic studies, it was postulated that Florida red tide potency *in situ* would be a complex phenomenon and the toxicologic consequences of red tides would be based on the amounts and activities of the brevetoxins present (Baden and Tomas 1988; Steidinger and Baden 1984).

**Figure 2:**
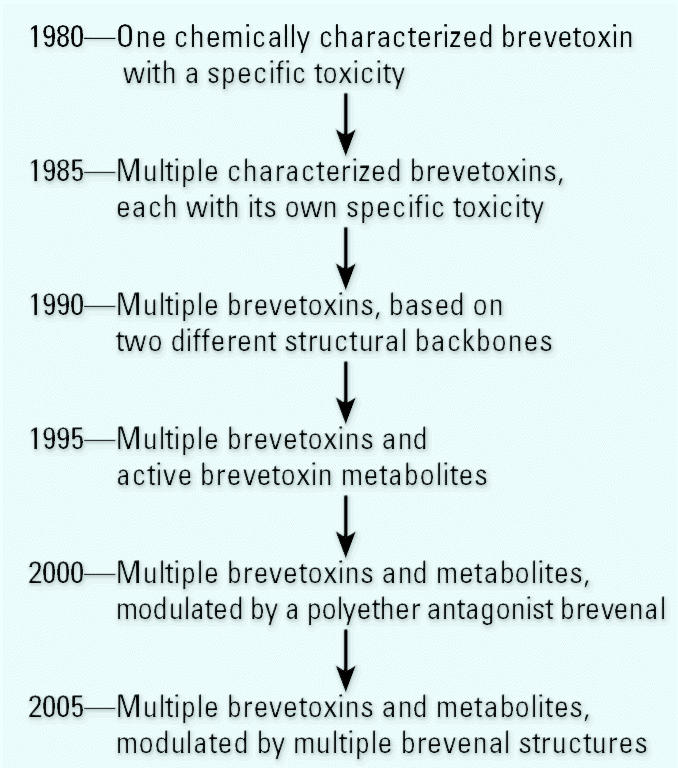
Complexity of lethal components isolated from Florida red tide: the explanation for the potency of red tide events is summarized from 1980 to the present. 1981: only one specific toxin had been identified. Lethality of red tides was thought to be directly related to the concentration of toxigenic organism and the toxin it produced. Mid-1980s: characterization of multiple toxins with widely varying potencies. Lethality was thought to arise from additive effects of each toxin present. Early 1990s: the second brevetoxin structural backbone (PbTx-1) and multiple derivatives discovered and thought to be more potent than the corresponding toxins based on the PbTx-2 backbone. Early 21st century: brevenal described as the first naturally occurring antagonist to counteract the effects of brevetoxins in radioligand binding assays, in fish and mouse bioassays, and in respiratory protocols. (The pulmonary receptor for brevetoxins and brevenal may be distinct from the well-characterized brevetoxin binding site on neuronal VSSC described in the text.)

The purpose of this mini-monograph introduction is to describe the complex nature of exposure to Florida red tide toxins, as reference for articles presented later in this monograph (Figure 2). Although most of the studies have been conducted with PbTx-3 (a natural toxin possessing all of the requisite structural characteristics for full activity), the investigators acknowledge that many studies will require repetition for toxins missing one or more activity loci, or for complex mixtures that may include metabolites and antagonists in addition to toxin. Presumably, any bioactive agents produced by the toxigenic organism or any metabolites free in the water have the potential to become aerosolized. Further, we believe that the overall signs and symptoms observed in humans will depend on the particular combination of activities elicited by the toxins and derivatives/metabolites (Jeglitsch et al. 1998).

We restrict ourselves to a discussion of those toxins, antagonists, and metabolites that have been described from seawater or culture medium, although we realize that aerosols may contain cell particles and organelles (Woodcock 1948) as well as bacteria, fungi, and other organic materials in the mixed microlayer of the coastal ocean (Zobell 1942). The metabolites produced by shellfish, fish, and other marine creatures, although extremely important for seafood poisonings, are not considered to be relevant to inhalation toxicology, and they have not been detected by us in air filters (Cheng et al. 2005).

**Figure 3:**
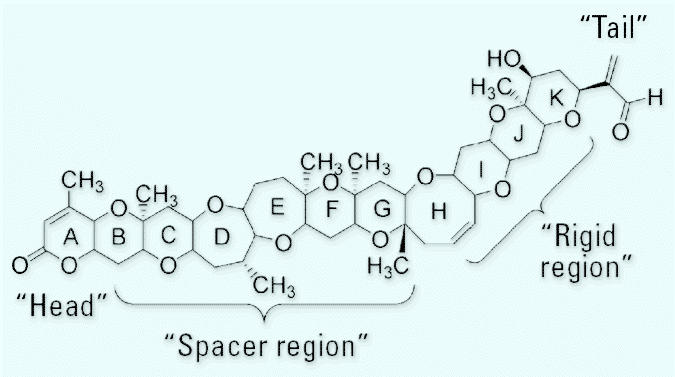
All active brevetoxins have several requisite features that result in complete expression of brevetoxin activities: a relatively rigid H–K ring region (G–J region in PbTx-1) thought to be involved in binding at site 5 of the VSSCs on the α-subunit (“rigid region”); an A-ring electrophile of some type, in most cases a five- or six-membered ring lactone (“head”); a several-ring “spacer” region that separates the binding region from the activity region; and a side chain (“tail”; Gawley et al. 1995).

Figure 3 illustrates the model “brevetoxin” that encompasses all the salient features of toxins that are “fully active” at their binding site, voltage-sensitive sodium channels (VSSCs; membrane potential-gated ion channels) (Baden et al. 1996). Modeling work initiated by Gawley et al. (1995) described a brevetoxin PbTx-2 molecule believed to be composed of three distinct segments: a relatively rigid fourring H–K ring system, an A-ring lactone, and a B–G ring region with limited flexibility. Homologous ring systems exist in the brevetoxin A backbone. All active toxins have these essential characteristics whether they are members of the brevetoxin B backbone or the brevetoxin A backbone (Figure 1).

**Figure 4:**
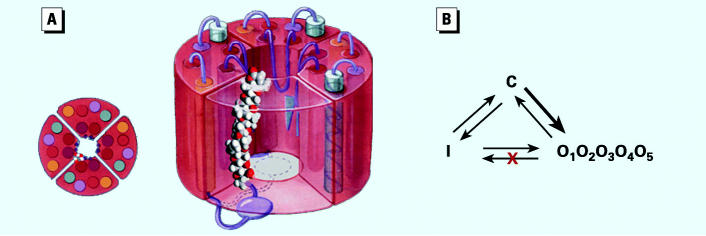
(*A*) A model depicting the sodium-channel α-subunit three-dimensional structure and hypothesized orientation of brevetoxin “head”-down” between domains III and IV. The view from above (at far left) illustrates a possible orientation relative to the α-helices, which are shown as colored circles. (*B*) Diagram illustrating the putative triad of VSSC configurations and alterations produced by brevetoxins. All natural brevetoxins *a*) shift the activation potential, favoring the open (O) configuration at normal resting potential (thick arrow); *b*) produce a longer mean open time (O); *c*) induce subconductance states (O_1_, O_2_, etc.); and *d*) inhibit inactivation (O→I). Figure adapted from Gawley et al. (1995), Taylor (1994), Rein et al. (1994a, 1994b), and Jeglitsch et al. (1998).

Active toxins bind to site 5 on the α-subunit of the VSSC (Poli et al. 1986) and are thought to orient “head-down” into the channel (Gawley et al. 1995), intercalating between the α-helices of domains III and IV of the VSSC (Trainer et al. 1994) (Figure 4).

The α-subunit is a single polypeptide glycoprotein, which possesses a 4-fold homology. Each homologous domain contains six trans-membrane α-helices (S1–S6), which alternately extend through the membrane, with short-to-long trans-helical peptide regions. Three of the helices in each domain are neutral in charge; two of the helices are hydrophobic, and one helix in each domain is highly positively charged. The highly charged S4 helix in each domain is thought to respond allosterically to changes in membrane potential, a “voltage sensor.” Allosteric realignment of all four S4 helices results in a change in channel configuration from “closed” (C), to “open” or conducting (O). Channels inactivate or become nonconducting (I) by internal insertion of the isoleucine–phenylalanine–methionine tripeptide region of the intra-cellular polypeptide connecting domains III and IV into the ion-conducting central pore. Normally, VSSCs open in response to membrane depolarization and subsequently inactivate in the late phase after activation. Channels return to the allosterically closed configuration during membrane repolarization (Taylor 1994). All natural brevetoxins have four distinct activities that alter the normal C→ O→ I triad of VSSCs: *a*) the activation potential is shifted to more negative potentials, favoring a C→O allosteric change at normal resting potential; *b*) a longer mean open time O, which can be influenced by a number factors, but which results in the channel being in the open configuration longer; *c*) an induction of sodium ion subconductance states in addition to the normal 21 pS rate; and *d*) an inhibition of inactivation O→I. Derivatives of natural toxin produced in the laboratory, as well as metabolites, knock out one or more of the observed activities in single ion channel patch-clamp experiments. The resulting overall consequences of exposure to multiple natural toxins and metabolites are complex and difficult to quantify mechanistically. How metabolites or modifications to toxin loci modulate activity is summarized in Table 1.

**Figure 6:**
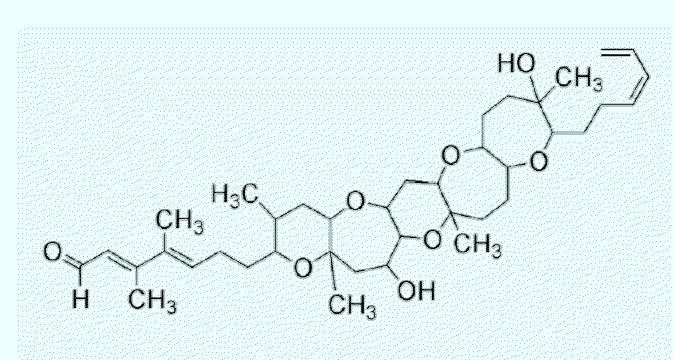
The brevenals are shorter *trans*-fused polyether molecules isolated from *K. brevis*. Consisting of a 6–7–6–7–7 ring motif, these materials bind with high affinity in brevetoxin receptor assays, and effectively act as inhibitors of brevetoxin binding and activity. Brevenal is the major constituent derived from cultures or the environment, with smaller amounts of brevenol (brevenal with the aldehyde reduced to the alcohol). Reduction of brevenal with tritiated borohydride can produce a new radioligand for probing polyether binding sites. Preliminary experiments indicate high-yield radioligand derivatization of brevenal. The final characterization of brevenal binding to determine if competition is truly competitive awaits completion.

PbTx-11, PbTx-12, and PbTx-tbm (brevetoxin PbTx-2 lacking the side chain tail) are new toxins found in cultures and in the field (Abraham et al. 2005; Bourdelais and Baden 2004; Bourdelais et al., unpublished results). Their toxicology is almost completely unknown except that they competitively bind at site 5 on the sodium channel (Bourdelais and Baden 2004; Bourdelais et al. 2004). Figure 6 shows the chemical structure of brevenal, the antagonistic material isolated from both laboratory cultures and bloom waters (Bourdelais et al. 2004, in press). Brevenal derivatives have also been detected in our cultures. Brevenal resembles hemibrevetoxin, isolated by Shimizu et al. (1990). Cytotoxicity was reported for hemibrevetoxin, but no antagonistic activity was recorded.

From the data collected, it is important to note again that not only are there two structural backbones for the brevetoxin molecules, but there are at least 13 different derivatives thereof, and each of these derivatives possesses a specific toxicity that is correlated to its binding affinity on VSSCs. However, from past work it is clear that derivatives produced at specific loci on the brevetoxin backbone impart a differential set of “activities” that encompass effects on activation potential, mean open time, inactivation, and persistent depolarization due to subconductance state prevalence.

## Relationship of Toxin Multiplicity to Environmental Episodes of Pulmonary Exposure

Many of the examined derivatives can be produced by intermediary metabolism and environmental decomposition, suggesting that environmental episodes may exhibit great variation in both degree and effect (Baden and Tomas 1988; Ingle 1954). The added presence of the brevenals would tend to moderate the overall consequences. Its waxing and waning presence during a red tide may provide a partial explanation for anecdotal accounts of high toxigenic organism cell counts in the absence of pronounced fish kills or respiratory irritation, or of relatively low cell counts and reports of “respiratory irritation” (Fleming et al. 2005).

What is there to say, then, about the overall pulmonary effects of these polyether natural products? First, in 1947 Woodcock collected droplets of vapor from active red tides and from normal seawater. The droplets from the red tide areas differed significantly from those from non-red tide areas (Woodcock 1948). Red tide aerosol droplets contained small, greenish granules similar in color and shape to those observed in the red tide organism (*K. brevis*). The granules, which were approximately 1–2 μm in diameter, are an ideal size to become delivery vehicles for pulmonary exposure. Evident from Woodcock’s work is not only that respiratory irritants become airborne from red tides but also that actual particles of the organism become airborne as well. Were these particles to be the delivery mechanism for at least part of the inhaled red tide brevetoxins, then it could be concluded that any toxin or metabolite present in the organism has the potential to be inhaled—perhaps as part of an intracellular storage granule or organelle. The search for the storage depot of brevetoxins in the *K. brevis* organism has been fruitless. Density centrifugation of whole *Karenia* cells indicates that the toxins are intracellular (Baden 1977). Density centrifugation of cell lysates fails to reveal any concentration of brevetoxins within any subcellular particle (Baden DG and Bishop B, unpublished observations). Nor has immunologic staining of the organism for toxin produced satisfactory results (Naar J, unpublished observations).

As described by Abraham et al. (2005), the brevetoxins have effects at 1,000-fold lower concentrations in pulmonary systems than they have in any neuronal system studied to date. Moreover, the relative concentrations of toxin and antagonist in the pulmonary system are reversed from those exhibited by neuronal systems. That is to say, brevenal is a more potent antagonist in pulmonary systems than it is in neuronal or fish bioassays. This is an interesting set of data because it tends to support the contention that there is something very different about the pulmonary receptor when compared with site 5 of VSSC in the central nervous system. Either way, further exploration of the binding phenomenon in pulmonary tissues is warranted.

The complex mixtures presented by Florida red tides (Cheng et al. 2005), all of which are known to bind to a single class of orphan receptors associated with VSSC site 5 (Gawley et al. 1992; Poli et al. 1986), provide insight into the neuronal effects of brevetoxins. That there are recognized effects in pulmonary systems (Abraham et al. 2005) and inhibitory effects on cathepsin catabolic enzymes (Sudaranam et al. 1992) only serves to illustrate the truly complex nature of the toxicology arising from the family of polyether natural products derived from *K. brevis*. Many of these effects are only now becoming clear. A nagging question is whether the pulmonary receptor is associated with the epithelial sodium channel (a ligand-gated ion channel) or if it is a new orphan receptor. Further, the inhibition of cathepsin within its active site by brevetoxin points to another potential activity for Florida red tide metabolites (Benson et al. 2005; Sudaranam et al. 1992).

We conclude that the toxicology surrounding exposure to Florida red tides involves multiple biotoxins and antagonists, and these interact with at least three receptors located in neuronal, pulmonary, and enzymatic regulatory systems of living organisms. The result is a complex combination of acute and chronic signs and symptoms in both animals and humans exposed to aerosolized bioactive materials produced by *K. brevis*.

## Figures and Tables

**Figure 5:**
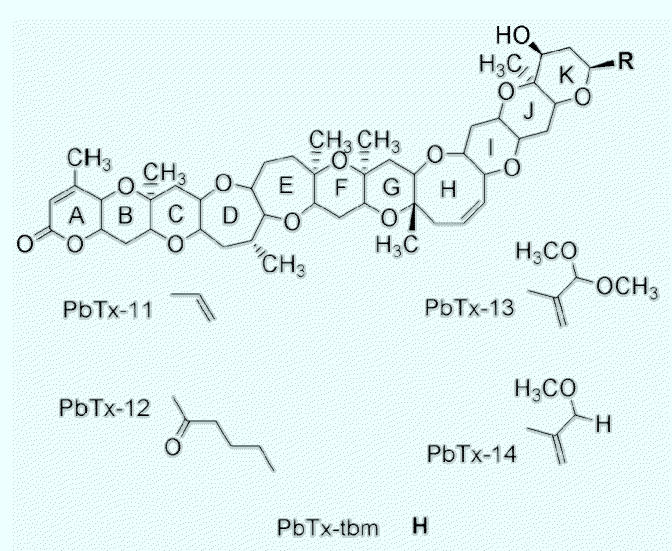
Five new brevetoxins, based on the PbTx-2 type backbone, have been purified and characterized: PbTx-11, a toxin with a shortened side chain; PbTx-12, the only natural ketone brevetoxin known; PbTx-13 and PbTx-14, which are both believed to be extraction artifacts formed in the presence of methanol reaction with the very active exomethylene-conjugated aldehyde of PbTx-2; and PbTx-tbm, a brevetoxin PbTx-2 backbone without any side chain, a form that is prevalent in senescent cultures.

**Table 1:** Effect of toxin modification on the five expressed activities of natural brevetoxins.*^a^*

Toxin modification	Shift activation	Subconductance states*^b^*	Longer mean open time	Inhibit inactivation	Toxin antagonist
Natural toxins	Yes	Yes (1)	Yes	Yes	No
Modified A-ring	Yes	No	No	Yes	No
Open A-ring	Yes	Yes (4)	Yes	Yes	No
Short spacer region	Yes	No	No	No	No
Bulky side chain	Yes	No	No	No	Yes
Saturated H-ring	No*^c^*	No	No	No	No
Brevenal	ND	ND	ND	ND	Yes

ND, not determined.

a Compiled from Baden et al. (1996), Bourdelais et al. (2005, in press), and Gawley et al. (1992).

b In addition to the normal 21 pS conductance.

c Has no affinity for site 5.
